# Creosote in Suppurative Nephritis

**Published:** 1898-05

**Authors:** 


### CREOSOTE IN SUPPURATIVE NEPHRITIS.

Dr. Leonard Weber, in some recent remarks on this subject before the Clinical Society of the New York Post-Graduate School, stated that he claimed priority for this special method of treatment. He had published his observations in this line in the latter part of 1893, and they had since been confirmed, and the method adopted by a number of other physicians. As inoperable cases of suppurative nephritis were not infrequently met with, and as he had observed such excellent results from the administration of creosote in pulmonary tuberculosis, he had been led to try it as a possible means of controlling the septic fever so commonly met with when the kidneys were the seat of suppurative process. His expectations had been fully realized, and not only did this drug greatly improve the patient's general condition, but it was not found necessary to increase the dose, as in pulmonary tuberculosis. He usually gave from 3 to 5 gr. of beechwood creosote, t. i. d., in capsules or alcoholic solution by mouth, or by the rectum in the form of suppositories or injections.

